# Polydopamine Nanomaterials: Recent Advances in Synthesis Methods and Applications

**DOI:** 10.3389/fbioe.2018.00109

**Published:** 2018-08-17

**Authors:** Vincent Ball

**Affiliations:** ^1^Faculté de Chirurgie Dentaire, Université de Strasbourg, Strasbourg, France; ^2^Unité Mixte de Recherche, Institut National de la Santé et de la Recherche Médicale, Strasbourg, France

**Keywords:** polydopamine, polydopamine nanoparticles, nanotubes, proteins, polyelectrolytes, surfactants

## Abstract

Polydopamine (PDA), the final oxidation product of dopamine or other catecholamines, attracted much attention as versatile coatings that can be used to cover the surface of almost all materials with a conformal layer of adjustable thickness ranging from a few to about 100 nm. These PDA layers can be subsequently modified with molecules carrying nucleophilic groups or with metallic nanoparticles from solutions containing metallic cations. However, during the deposition of PDA film on the surfaces, the reaction products that are simultaneously obtained from the oxidation of catecholamines in solution precipitate. Hence, some recent effort has been devoted to produce PDA in the form of nanoparticles. The aim of this short review is to give a comprehensive description of the synthesis methods yielding of PDA nanoparticles in the absence or in the presence of templating agents (polymers, polyelectrolytes, surfactants, proteins, and even some small organic molecules). We will also describe the use of thin PDA layers to coat already synthesized nanoparticles or nanotubes. Finally, several first applications of the obtained PDA nanoparticles will be described.

## Oxidation of catecholamines and its consequences on surfaces and in solution

**Scheme 1:**
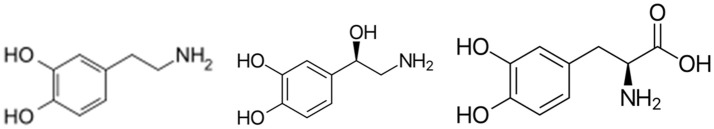
Characteristic structure of catecholamines: dopamine, norepinephrine, and L-DOPA (from left to right).

During the whole of the Twentieth century, guided by technological requirements, surface science was characterized by the development of surface modification methods, such as the protection against corrosion and the development of anti-adhesive coatings. It appeared that each kind of material required some specific functionalization chemistry. For instance, the noble metals can be functionalized by covalent bonds using alkanes modified with thiols (Ullmann, 1996). The oxides can be modified with silanes or phosphonates (Sagiv, 1980). The surface of polymer materials can be modified if the polymers carry reactive lateral side chains, but the chemical modification of the surface of polymeric materials can remain a challenge. The layer-by-layer deposition of polyelectrolyte multilayer films offers an interesting functionalization method of charged surfaces (Decher, 1997; Borges and Mano, 2014; Richardson et al., 2016) but it is not applicable on a number of materials. Moreover, the obtained coatings, even if their thickness can be finely tuned (by altering the number of deposition steps or the physicochemical parameters of the solution), are often not stable enough for long-term applications (though chemical crosslinking may reinforce these coatings). This is to say that the development of versatile coating methods that are able to functionalize on a vast repertoire of material surfaces, appearing possible with the development of catecholamine-based coatings, was a major advance in surface science (Lee et al., 2007; Kang et al., 2009; Hong et al., 2013). These coating methods rely on the oxidation of catecholamines, such as dopamine, norepinephrine, and L-DOPA (Jaber and Lambert, 2010), and their subsequent polymerization or self-assembly to a eumelanin-like material called “polydopamine” (PDA). The whole surface functionalization strategy relies on biomimetism: the proteins on the extremity of the mussel byssus are extremely rich in L-DOPA (up to 30 mol%) and L-Lysine residues (Lee et al., 2011). These specific amino acid residues, containing a catechol and an amine as functional groups in L-DOPA and L-Lysine, respectively, allow for a strong adhesion of the mussel to all kinds of substrates in a wet and slightly basic environment of sea water. The rationale behind the use of catecholamines to deposit the adhesive coatings on all kinds of materials was the simultaneous presence of a catechol and an amine functional group (Scheme 1).

**Figure 1:**
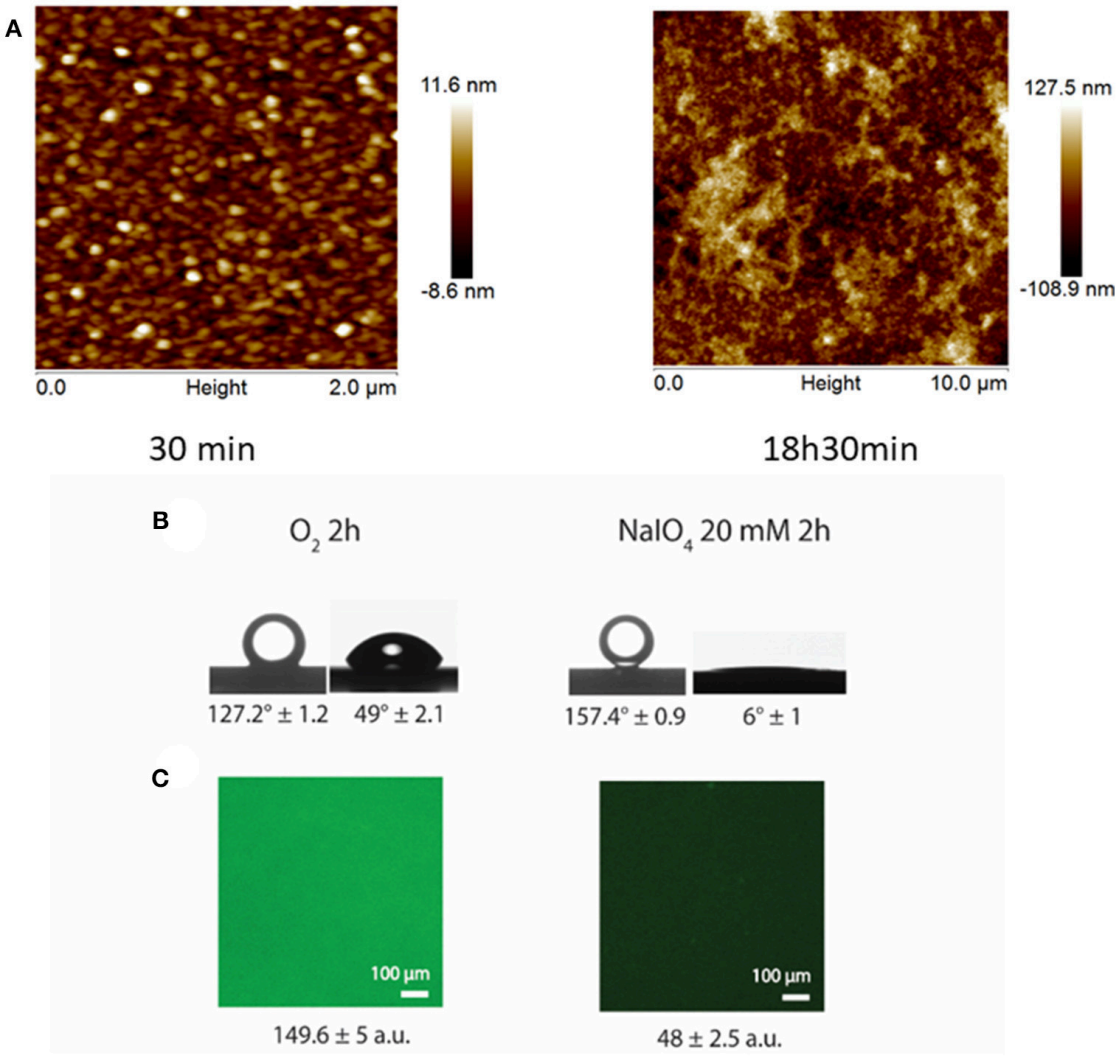
**(A)** AFM surface topographies of PDA-NaIO_4_-20mM films on silicon wafers as a function of the reaction time. **(B)** Underwater contact angles of CHCl_3_ (left) and water contact angles (right) for PDA-O_2_-2 h (left) and PDA-NaIO_4_-20 mM-2 h (right) films. **(C)** Adsorption of BSA-FITC on PDA-O_2_-2 h (left) and PDA-NaIO_4_-20 mM−2 h (right). Reproduced from Ponzio et al. (2016) with authorization.

Most catecholamine-based coatings are produced from dopamine in the presence of Tris buffer at pH = 8.5, by using O_2_ dissolved in water as the oxidant. However, it was soon found that several other oxidants can be used as well, such as ammonium peroxodisulfate and sodium periodate (Wei et al., 2010), or UV irradiation to generate free radical species (Du et al., 2014). The used oxidant has a major impact on the kinetics of film deposition and on the film composition and structure. For instance, using sodium periodate as the oxidant leads to fast film growth with the possibility for the film to reach a thickness close to 100 nm, in about 2 h, in the presence of 2 mg mL^−1^ dopamine and 10 mM NaIO_4_ (Ponzio et al., 2016). On the other hand, in the presence of Tris buffer and O_2_ as the oxidant, the film thickness saturates at 40–45 nm after about 16 h of oxidation (Lee et al., 2007; Ponzio et al., 2016). The hydrophilic character of the PDA-NaIO_4_ films is markedly improved with respect to the PDA-O_2_ films (Figure 1).

There are therefore many kinds of PDA films depending on the synthesis conditions (d'Ischia et al., 2014), and great care should be taken to describe the reaction conditions when dealing with the applications of such films. Some alternative methods to dipping the substrate in the aqueous solution were recently developed to deposit PDA and PDA-like films from organic solvents (You et al., 2017) or from the solid phase (Pezzella et al., 2014) or by spray deposition (Schlaich et al., 2018).

The PDA films can also be deposited at the water/air interface and transferred therefrom to solid substrates by the Langmuir–Schaeffer method (Ponzio et al., 2014b). Such films are nevertheless extremely brittle. They can be consolidated from a mechanical point of view using poly(ethylene imine) (Hong et al., 2014; Yang et al., 2014) or alginate modified with catechol groups (Ponzio et al., 2017) to yield extremely thick (hundreds of nanometers), flexible, anisotropic, and humidity-responsive membranes.

**Scheme 2:**
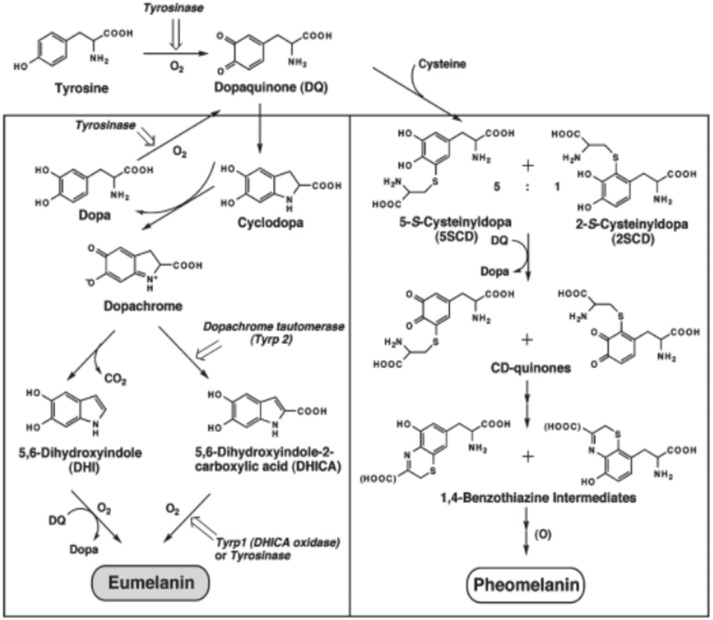
Chemical pathways leading to eumelanin (left column) and pheomelanin (right column) in living organisms. The first enzymatically catalyzed step is omitted in the synthesis of synthetic melanins. Reproduced from Ito (1989), with authorization.

Even if there is a major uncertainty about the molecular structure of PDA and the related materials being either a polymer or a supramolecular aggregate, some strong arguments show the presence of non-covalently bound small oligomers of dopamine (Hong et al., 2012; Ponzio and Ball, 2014). The overall structure and properties of PDA and the related materials produced from the oxidation of catecholamines are very close to those of eumelanins, the black-brown pigment of the skin and hairs (Meredith and Sarna, 2006; d'Ischia et al., 2009; d'Ischia et al., 2014). The known chemical pathways leading to the brown-black eumelanin and the reddish pheomelanin are summarized in Scheme 2.

When PDA is produced using the oxidants added in the solutions, the PDA films are deposited on the walls of the reaction beakers and on the substrates of interest, but some insoluble black precipitate, forming the main reaction product, is deposited at the bottom of the reaction vessel. This is a major drawback which can be avoided when dopamine is electropolymerized on the surface of a conductive material (Li et al., 2006; Bernsmann et al., 2011; Vatral et al., 2015). Unfortunately, only a low proportion of substrates are conductive and hence, interest has been shown to valorize PDA produced in solution. It is the aim of this article to summarize the research involved in producing PDA particles of controlled size with or without the use of templating agents. Some attention will also be given to the use of PDA to coat already formed nanoparticles or nanotubes.

### Polydopamine nanoparticles without templating agents

**Figure 2:**
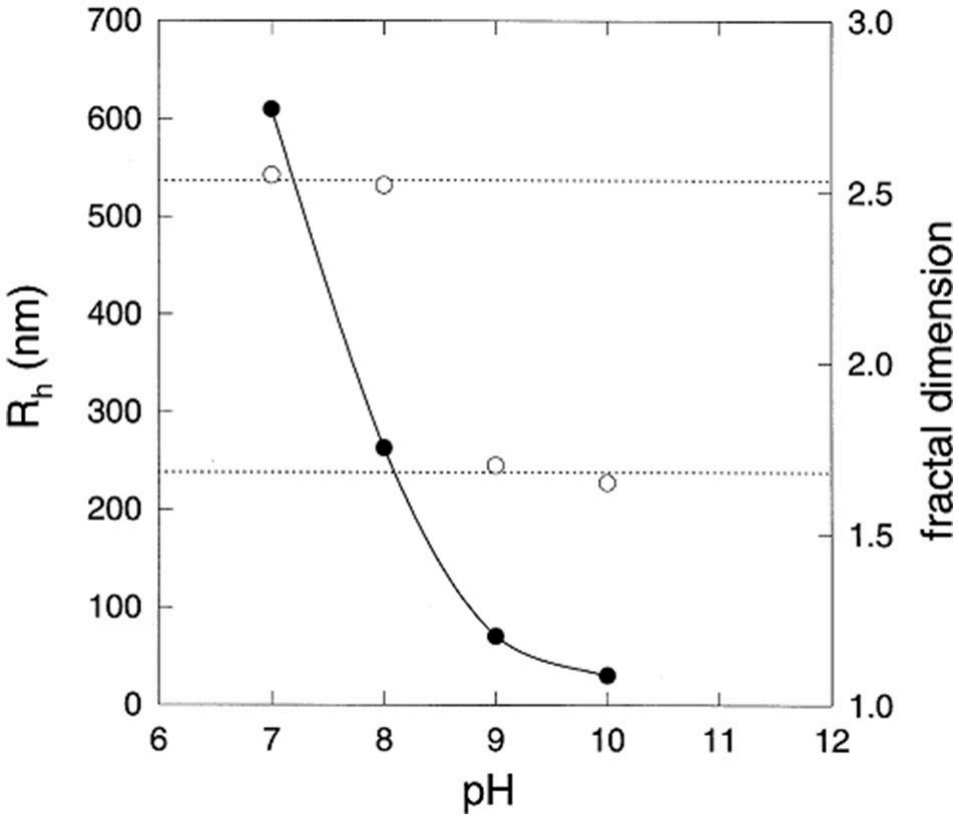
Hydrodynamic radius (left hand vertical scale, 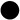) and fractal dimension (right hand vertical scale, 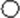) of eumelanin grains obtained at the end of the reaction kinetics from L-DOPA solutions, as function of the pH. Reproduced from Bridelli (1998) with authorization.

Interestingly, the eumelanin grains synthesized from L-Dopa show a pH-dependent size and a pH-dependent fractal dimension. The size of these nanoparticles decreases with an increase in the pH, with the possibility to reach the range of tens of nanometers at pH 10 (Bridelli, 1998) (Figure 2).

In analogy with the Stöber process to synthesize silica nanoparticles from silicon alkoxydes via hydrolysis and condensation in ethanol-water mixtures and in the presence of ammonia as a catalyst, the PDA nanoparticles in the diameter range of 100 nm and 200 nm could be obtained (Ju et al., 2011). In these synthesis protocols, a 90 mL dopamine hydrochloride solution at 2 mg.mL^−1^ was titrated with 760 μL of 1 M NaOH and reacted in the presence of dissolved O_2_ for 5 h at 50°C. The large-sized aggregated material was discarded by centrifugation at 4,000 rpm and only the suspension of smaller particles was investigated (Ju et al., 2011). Even if this purification step is time consuming, the eumelanin spheres with a diameter of (84 ± 16) nm were characterized by transmission electron microscopy. The particle size was found to decrease with a temperature increase of the reaction medium up to 70°C. The colloidal stability of these nanoparticles in the presence of phosphate buffer and fetal bovine serum was markedly increased after the grafting of methoxy-poly(ethylene glycol)-thiol (m-PEG-SH) forming monolayers on the surface of PDA. This synthesis method was further adapted by Amin et al. (2017) and the obtained nanoparticles were made fluorescent either by adding rhodamine 123 or rhodamine B to the dopamine solution or by adding the dye to the m-PEG-SH capped PDA nanoparticles (Amin et al., 2017).

The synthesis of PDA nanoparticles was further improved with the use of water-ethanol mixtures by adding ammonia as a catalyst in the dopamine containing solvent (Liu et al., 2013; Wang et al., 2016).

Very small PDA nanoparticles, with diameters of only a few nanometers, can be synthesized by anodic microplasma electrochemistry (Wang et al., 2018). In this synthesis method, the dopamine is oxidized by the plasma-generated reactive oxygen species at the solution/air interface. This process also produces an acidification of the solution (final pH = 5, where normally the dopamine is not auto-oxidized by the dissolved oxygen) limiting the further aggregation of the obtained nanoparticles. Prolonged microplasma treatment of the solution/air interface then induces an increase in the concentration of monodisperse nanoparticles (Wang et al., 2018). The obtained PDA nanoparticles are fluorescent with maximal emission occurring at around 440 nm when the excitation wavelength was set at 360 nm. The quantum yield of these fluorescent PDA nanoparticles was equal to 0.58% using quinine sulfate as a standard. This fluorescent quantum yield is surprisingly high for a eumelanin-like nanomaterial (Meredith and Sarna, 2006) and may well reflect the very small diameter of the obtained nanoparticles, which is 3.1 nm as obtained by transmission electron microscopy.

Similar PDA nanoparticles with excitation wavelength-dependent emission spectra have been described (Zhang et al., 2012; Yildirim and Bayindir, 2014). On the other hand, the excitation wavelength-independent emission could be obtained when dopamine was oxidized in the presence of glutaraldehyde (for 12 h), then reduced with NaBH_4_ (for 2 h), and finally brought in contact with ammonium hydroxide and mercaptoethanol (for 30 min) (Xiong et al., 2014). The obtained dopamine oligomers contained 5 dopamine units on an average, and the maximum emission occurred at λ = 485 nm for all excitation wavelengths between 360 and 440 nm. The quantum yield of these oligomers was as high as 16.2% using quinine sulfate as a standard. In addition, the obtained dopamine oligomers were photostable in marked contrast to most of the commercially available organic fluorescent molecules (Xiong et al., 2014).

The Fe^3+^ cations can be directly incorporated in the PDA nanoparticles (Li et al., 2016) and pre-incorporated Mn^3+^ cations can be exchanged for Gd^3+^ cations in the PDA nanoparticles of 160 nm in diameter (Wang et al., 2017). Similarly, PDA hollow nanotubes could be obtained on curcumin cylindrical crystals. This approach is particularly interesting because the curcumin solid templates were obtained by phase separation upon addition of the aqueous phase (Tris buffer at pH = 8.5) to an acetone/ethanol mixture (1:1 v/v) containing the curcumin/dopamine mixture. In such a solvent, curcumin is soluble and dopamine is stable. The addition of water induces the crystallization of curcumin and the oxidation of dopamine to produce PDA. The curcumin cores can be dissolved again by washing the composite material with ethanol (Xue et al., 2016). A similar concept has been applied using ZnO nanorods, having a diameter of 60 nm, to produce hollow PDA nanotubes with a wall thickness increasing from 13 to 75 nm when the oxidation time of dopamine (1 mg.mL^−1^ in the presence of 10 mM Tris buffer at pH = 8.5) is increased from 24 to 120 min (Yan et al., 2016).

Of course, the already formed nanoparticles can be coated with thin PDA films. This concept has been applied to produce hollow PDA capsules (Postma et al., 2009) after dissolution of the core or to produce hydrid core shell nanoparticles. As another interesting example, Fe_3_O_4_ nanoparticles were successively coated with a gold layer, a 4 nm thick PDA coating onto which folic acid was grafted using standard carbodiimide chemistry (Li H. et al., 2017). The grafted folic acid allowed to target cancer cells which are rich in folic acid receptors.

Tip-sonication of a dopamine-hydrochloride solution containing AgNO_3_ or CuSO_4_ at the location of the solution/n-decane interface produces metal-composite PDA nanoparticles. Carrying out sonication (150 W.cm^−2^ at 20 kHz) for 6 min produces Cu-PDA nanoparticles and Ag-PDA nanoparticles having a diameter of (259 ± 31) nm and (295 ± 38) nm, respectively. On using a mixture of CuSO_4_ and AgNO_3_ during the synthesis, the obtained particles had an average diameter of (270 + 28) nm after the same sonication step (Yeroslavsky et al., 2016). In these particles, metallic silver was located in the core whereas copper, mostly in its +II oxidation state, was located at the shell of the particles.

Finally, the hydroxyl radical induced degradation of the already formed polydopamine produces extremely small (with diameter in nanometers) fluorescent PDA-based dots (Lin et al., 2015).

## Polydopamine nanoparticles and nanotubes with templating agents

**Figure 3:**
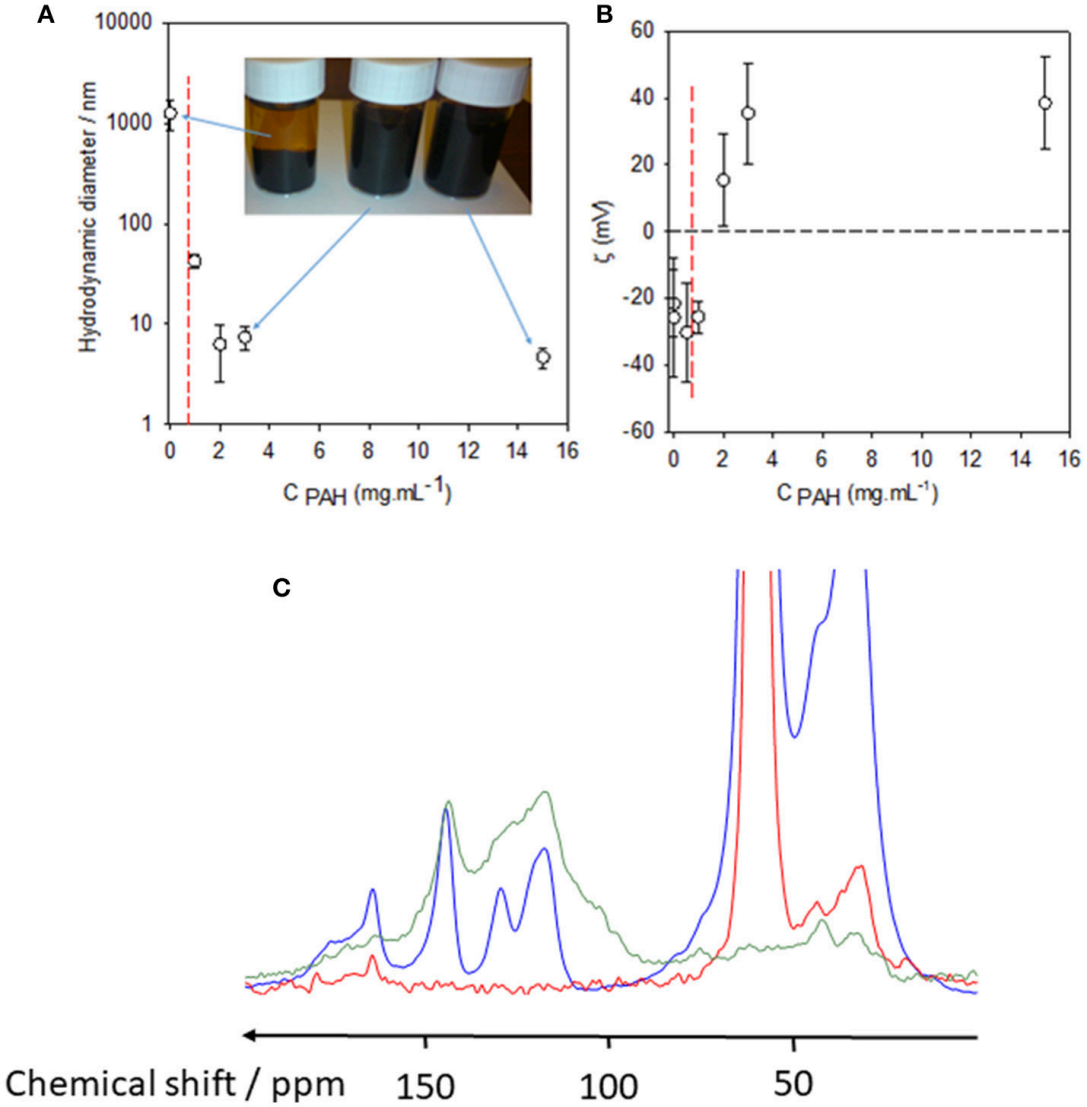
Hydrodynamic diameter **(A)** and zeta potential **(B)** of PDA particles obtained after 24 h of dopamine oxidation (2 mg.mL^−1^ in the presence of 50 mM Tris buffer at pH = 8.5) as a function of the PAH concentration in the reaction medium. The error bars correspond to one standard deviation on the mean (*n* = 10 measurements). The black dashed line in **(B)** corresponds to the zero value of the zeta potential and is aimed to guide the eye. The vertical red dashed line corresponds to the PAH concentration (0.7 mg.mL^−1^) above which the PDA particles remain stable, without phase separation, even after prolonged storage. The inset in **(A)** corresponds to a picture of the PDA containing solutions taken 7 days after the completion of the oxygenation of the dopamine solutions in the presence or in the absence of PAH as indicated with blue arrows. **(C)** ^13^C CP-MAS Spectra of the PAH-only sample (red curve), PAH-PDA (blue curve), and pristine PDA (green curve). The PAH concentration was 2 mg.mL^−1^ in the synthesis batch as well as in the reference PAH solution. Data have been taken from Mateescu et al. (2016) with authorization.

When considering the size distribution of eumelanin grains in the skin, it immediately appears that they are constituted from aggregates of uniform nanoparticles of 100–200 nm, which in turn are constituted from smaller aggregates (Clancy and Simon, 1998). In addition, these nanoparticles are always surrounded by a protein capping layer (Guo et al., 2008). It was also found that some proteins present in the melanosomes accelerate the polymerization of eumelanin from tyrosine by abstracting some protons from the monomers, hence accelerating the oxidation of L-DOPA, which is the first step in the complex chemical pathway leading to eumelanin (Scheme 2) (Wagh et al., 2000). These findings incited the investigation of the formation of PDA in dopamine solutions containing synthetic polymers (Arzillo et al., 2012), polyelectrolytes (Mateescu et al., 2016), or proteins (Chassepot and Ball, 2014). It was found that poly(vinyl alcohol) limits the size of PDA to about 100 nm (Arzillo et al., 2012). Similarly, the polycations such as poly(allyl amine hydrochloride) (PAH) produce PDA nanoparticles as small as 10 nm (as characterized by means of dynamic light scattering) when the polyelectrolyte concentration is greater than 2 mg.mL^−1^ after 24 h of oxidation in the presence of air (O_2_ being the oxidant) and at pH = 8.5 (Figure 3A) (Mateescu et al., 2016). The critical polyelectrolyte concentration to obtain a stable colloidal suspension of PDA particles was found to be 0.7 mg.mL^−1^ (about 8 mM in monomer units) in the presence of dopamine at 2 mg.mL^−1^ (10.6 mM). Above this critical concentration the zeta potential of the particles becomes positive, whereas the pristine PDA nanoparticles (in the μm size range) have a negative zeta potential at the same pH value (Figure 3B). The ^13^C-MAS NMR spectroscopy has shown that the peaks of the PDA capped with PAH are thinner than those of the PDA obtained in the same conditions but without the polyelectrolyte. Other polycations such as poly(diallyldimethylammonium chloride) (PDADMAC) also control the size of the obtained PDA nanoparticles. However, in the latter case the used quaternary amine-based polycation cannot interact with the PDA through covalent binding occurring via nucleophilic addition of amines to quinone groups. Instead, the PDADMAC interacts with the negatively charged PDA through electrostatic interactions (Ball, 2010). Surprisingly, the polyanions such as poly(4-styrene sulfonate) and poly(acrylic acid) reduce the size of the PDA particles in a polyelectrolyte concentration-dependent manner (Mateescu et al., 2016). In all the cases, the reduction in the size of the PDA nanoparticles with the polyelectrolyte concentration is accompanied with a progressive inhibition of the deposition of PDA on the walls of the reaction beaker, thereby implying that free dopamine and oligomers thereof are not available anymore to bind on the solid/liquid interface when the polyelectrolyte concentration is above a critical, polyelectrolyte-dependent threshold value.

**Figure 4:**
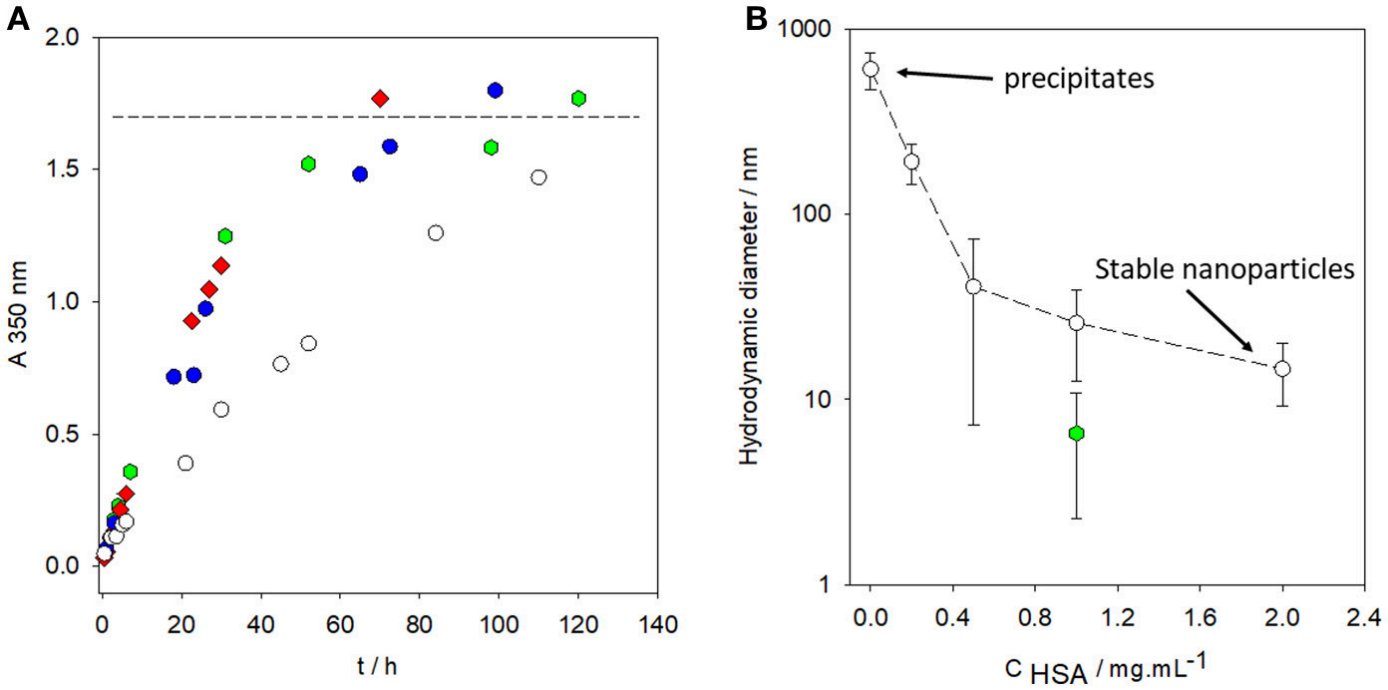
**(A)** Absorbance at λ = 350 nm vs. time for dopamine solutions placed in oxidizing conditions (in the presence of O_2_ from the air and at pH = 8.5, 50 mM Tris buffer) in the absence of HSA (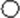) and in the presence of HAS at various concentrations: 0.2 mg.mL^−1^ (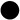), 1 mg.mL^−1^ (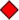), and 2 mg.mL^−1^ (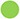). The horizontal dashed line corresponds to the saturation absorption at the end of the oxidation kinetics of dopamine. **(B)** Hydrodynamic diameter of PDA particles synthesized for 24 h from a 2 mg.mL^−1^ dopamine solution (50 mM Tris buffer at pH = 8.5) in the presence of HSA at different concentrations (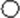). Hydrodynamic diameter of PDA particles prepared in the same conditions as previously described and stored in a closed bottle (without refreshed air) for 3 months before characterization by dynamic light scattering (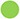). Reproduced from Chassepot and Ball (2014) with authorization.

Proteins such as human serum albumin (HSA) speed up the production rate of PDA in solution, as evaluated by means of UV-vis spectroscopy (Figure 4A), and reduce the hydrodynamic diameter of the PDA nanoparticles after 24 h of oxidation in the presence of 50 mM Tris buffer at pH = 8.5 (dissolved O_2_ being the oxidant) (Figure 4B). Similar to polycations (Mateescu et al., 2016), in the presence of a threshold concentration in HSA, the deposition of PDA on the surface of the reaction beaker is totally inhibited (Chassepot and Ball, 2014).

Some proteins, such as bovine pancreatic alkaline phosphatase, allow for a similar protein concentration-dependent control in the size of PDA nanoparticles whereas other proteins (such as hen egg white lysozyme and bovine α-lactalbumin) do not control the size of the PDA aggregates (leading to precipitation after a few hours of oxidation) and do not impede the deposition of PDA films on solid/liquid interfaces. The reason why some proteins control the formation of well-defined colloidal PDA nanoparticles, while some others do not influence the oxidative self-assembly process, remains to be investigated from a fundamental point of view.

Smaller molecules, such as folic acid (Yu et al., 2014) and DNA origamis (Tokura et al., 2018), were found to have a control over the size and morphology of PDA assemblies. In particular, folic acid (0.15 mg.mL^−1^) and dopamine (0.3 mg.mL^−1^) were mixed for 1 day before the addition of Tris buffer (10 mM, pH = 8.8). Nanofibers were formed preferentially at 60°C and in the dark in order to protect the folic acid from photodegradation. The yield in nanofibers increased between 1 and 30 h of oxidation. Some arguments, based on the analysis of atomic force microscopy images, were given to suggest that the nanofibers were formed from nanobelts or nanosheets undergoing a curling process. Folic acid is supposed to favor the formation of these nanobelts and nanosheets through π stacking with small self-assembled aggregates of the oxidation products of dopamine (Yu et al., 2014).

**Figure 5:**
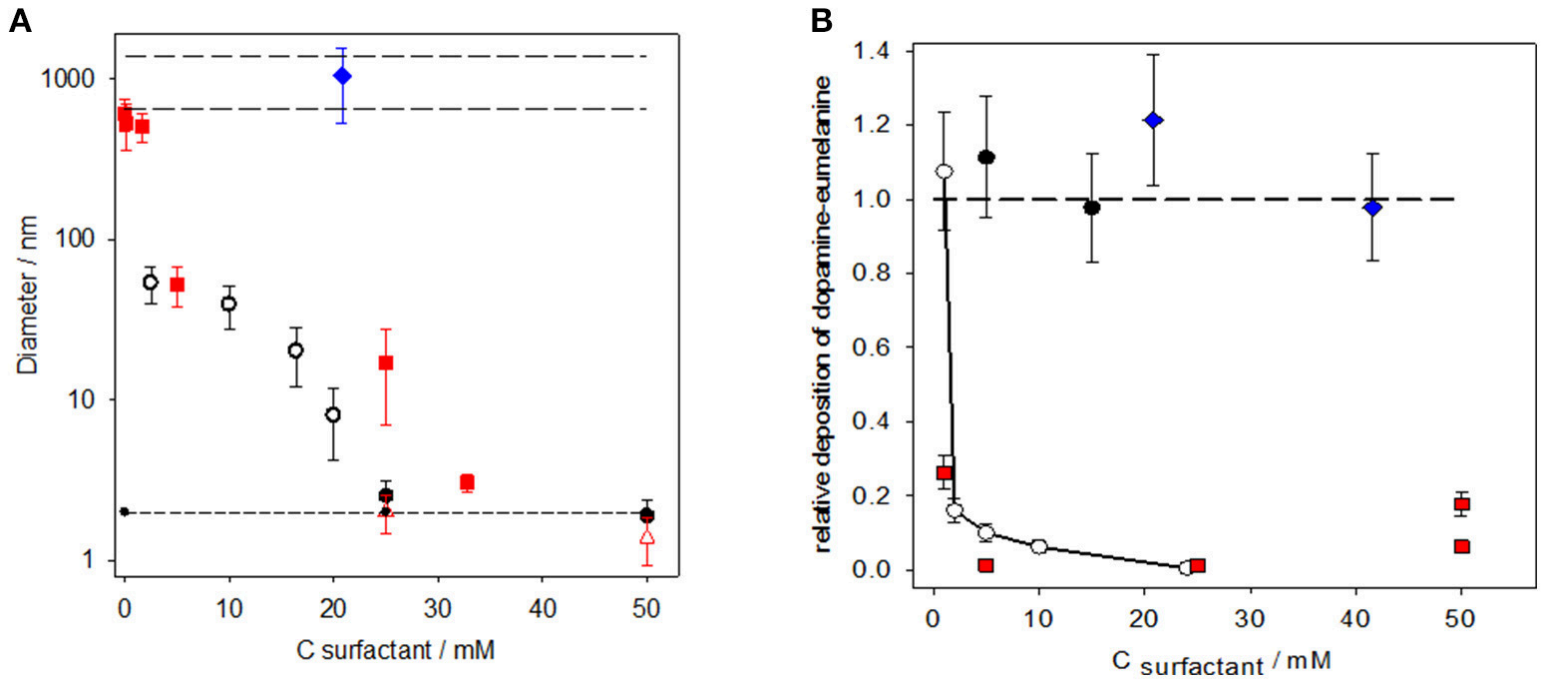
**(A)** Evolution of the hydrodynamic diameter of PDA aggregates as a function of the surfactant concentration in the case of SDS (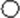), HTAB (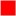), and Triton X-100 (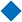). The dopamine concentration was 2 mg.mL^−1^ in the presence of 50 mM Tris buffer at pH = 8.5 in all experiments. The long-dashed lines in the upper part of the figure correspond to the size of the PDA prepared in the absence of surfactant, whereas the short-dashed lines in the lower part of the figure corresponds to the size of the surfactant micelles [measured in the case of SDS (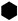) and HTAB (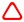)]. **(B)** Relative deposition of PDA films as calculated by dividing the thickness of the PDA films, obtained on silicon in the presence of a surfactant, by the film thickness obtained in the absence of a surfactant. The film thickness was obtained by means of single wavelength ellipsometry fixing the complex refractive index of PDA to 1.73 ± 0.02i at λ = 632.8 nm. The deposition of PDA on silicon was investigated in the presence of different concentrations of SDS (^___
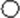
___^), HTAB (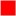), sodium octylsulfate (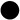), and Triton X-100 (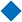). The error bars are calculated from the standard errors on the film thickness produced both in the absence and presence of surfactants. The long-dashed line has the same significance as in **(A)**. Reproduced from Ponzio et al. (2014a) with authorization.

It has also been found that the surfactants, such as sodium dodecyl sulfate (SDS) and hexadecyltrimethylammonium bromide (HTAB), enable the acceleration of the oxidation process of dopamine and a progressive reduction of the size of the obtained PDA aggregates. When the surfactant concentration is increased, these PDA particles finally reach a hydrodynamic diameter that is slightly larger than the hydrodynamic diameter of the surfactant micelles obtained above the critical micellar concentration of the surfactant (Figure 5A) (Ponzio et al., 2014a). Note also that the surfactants that are able to control the size of the PDA particles are also efficient in reducing the thickness of the PDA coatings deposited on the surface of the silicon slides immersed in the dopamine + surfactant solutions (Figure 5B).

It was also found that some uncharged surfactants (such as Triton X100) and negatively charged surfactants with shorter alkyl chains (such as octyl sulfate) do not control the formation of PDA (Figure 5).

The triblock F127 copolymer with 1,3,5-trimethylbenzene (TMB) incorporated in the hydrophobic propylene oxide core of the micelles produced mesoporous PDA particles about 90–100 nm in diameter with a porous size distribution depending on the TMB/F127 ratio after the removal of the F127-TMB core (Chen et al., 2016).

Overall, the main synthesis strategies of PDA nanomaterial synthesis, either with or without templates, are summarized in Table 1.

**Table 1:** Summary of the major synthesis protocols of PDA nanomaterials.

**Main idea of the PDA nanomaterial synthesis**	**With or without templates and average size of the obtained nanomaterials**	**References**
Water/ethanol mixtures Ammonia as a catalyst	Without template 100–200 nm	Ju et al., 2011 Liu et al., 2013; Wang et al., 2016
Microplasma electrochemistry at the water/air interface	Without template About 3 nm	Wang et al., 2018
Dopamine, glutaraldehyde (for 12 h), reduction with NaBH_4_ (for 2 h), finally ammonium hydroxide and mercaptoethanol (for 30 min)	Without template Only a few dopamine units	Xiong et al., 2014
Incorporation of metallic actions in the PDA nanoparticles	Without template About 100–200 nm	Li et al., 2016
PDA deposition on sacrificial nanorods or microparticles	Wall thickness of PDA between 13 and 75 nm	Postma et al., 2009; Xue et al., 2016; Yan et al., 2016
Hydroxyl radical induced degradation of larger polydopamine	Without template A few nanometers in diameter	Lin et al., 2015
Tip sonication at the aqueous solution/n-decane interface	Without template Between 250 and 300 nm with incorporated metals	Yeroslavsky et al., 2016
Dopamine oxidation in the presence of synthetic polymers, polyelectrolytes	With templates Tens to hundreds of nanometers	Arzillo et al., 2012; Mateescu et al., 2016
Dopamine oxidation in the presence of specific proteins	With templates Diameter depending on the initial protein/dopamine ratio	Chassepot and Ball, 2014
Dopamine oxidation in the presence of DNA origamis	With templates A few nanometers in diameter	Tokura et al., 2018
Using π stacking interactions with folic acid, optimal reaction temperature at 60°C	With templates Obtention of PDA nanofibers	Yu et al., 2014; Fan et al., 2015
Dopamine oxidation in the presence of surfactants or block copolymer micelles	With templates Possibility to use the size of the template to control the diameter of the PDA-based material	Ponzio et al., 2014a, Chen et al., 2016

## Applications of polydopamine nanoparticles

The applications of PDA nanoparticles are by far less developed in comparison to those of PDA-based films owing to the recent development of the synthesis methods of these nanoparticles as described in sections Polydopamine Nanoparticles Without Templating Agents and Polydopamine Nanoparticles and Nanotubes with Templating Agents.

### PDA nanoparticles in thin films

PDA nanoparticles with a diameter of (146 ± 15) nm were synthesized in a water-ethanol mixture in the presence of ammonia and self-assembled via the vertical evaporation technique to obtain films displaying structural colors (Xiao et al., 2015).

**Figure 6:**
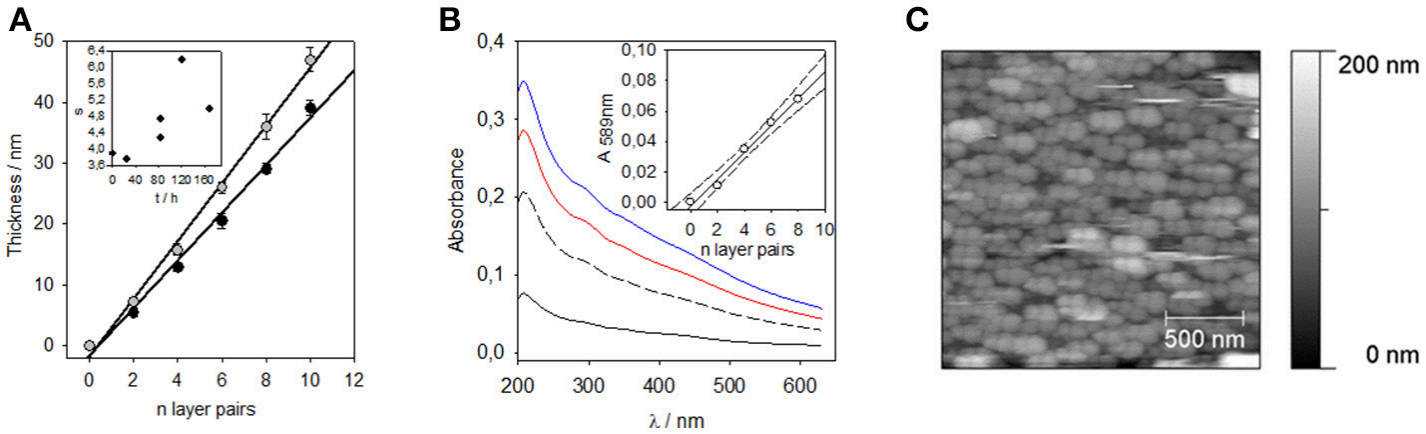
**(A)** Thickness of (PDADMAC-PDA)_n_ multilayer films deposited on silicon slides as measured by single wavelength ellipsometry (circles) and linear regressions to the data (lines). The error bars represent one standard deviation over 5 measurements taken on the same sample. (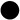), Films deposited immediately after the end of the 24 h dopamine oxidation (2 mg.mL^−1^ at pH = 8.5), (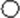), film deposited after 3.5 days of aging of the PDA suspension. Inset: Slope of the thickness vs. the number of layer pairs as a function of the aging time of the PDA suspension before deposition of the (PDADMAC-PDA)_n_ film. **(B)** UV-visible spectra of the (PDADMAC-PDA)_n_ films for *n* = 2 (black solid line), *n* = 4 (black long-dashed line), *n* = 6 (red solid line), and *n* = 8 (blue solid line). For these experiments, the PDA containing suspension was aged for 24 h before the film deposition. The inset displays the absorbance at λ = 589 nm as a function of the number of deposited layer pairs. The straight line and the dashed lines correspond to a linear regression to the data and to the limits of the 95% confidence interval, respectively. **(C)** AFM topographies of a (PDADMAC-PDA)_10_ film prepared using a PDA containing solution aged for 3 days before film deposition. Reproduced from Bernsmann et al. (2010) with authorization.

PDA-based particles can be deposited in alternation with polycations, such as poly (diallyldimethyl ammonium chloride) (PDADMAC), using the layer by layer deposition method (Decher, 1997; Borges and Mano, 2014; Richardson et al., 2016) (Figure 6A) owing to their negative surface charge at a pH greater than 4.5 (Ball, 2010). The absorption spectra of these (PDADMAC-PDA)_n_ films (where *n* is the number of adsorption cycles from PDADMAC and PDA containing solutions) were similar to the absorption spectra of pure PDA-based films (Figure 6B) featuring the typical broadband absorption of eumelanin-like materials. The absorbance scaled linearly with the film thickness (Inset in Figure 6B) and the films obtained from the layer-by-layer deposition process were constituted of particles having an average diameter of 300–400 nm (Figure 6C).

### PDA nanomaterials as a sensing platform

An emerging trend is the use of PDA nanoparticles as sensors. PDA by itself, owing to its absorption spectrum covering the whole UV-visible and near infrared range, is an extremely efficient fluorescence quencher, for dyes including aminomethylcoumarin acetate 6-carboxyfluoresceine, 6-carboxytetramethylrhodamine, and Cy5. When such dyes are bound either at the 5′ or 3′ end of a single stranded DNA, the DNA loses its fluorescence upon binding to the PDA nanoparticles (either 336 nm or 54 nm in diameter), but the fluorescence is recovered in a dose-dependent manner when the DNA bound nanospheres are placed in contact with the complementary DNA strands (Qiang et al., 2014). The detection limit of these sensors can be as low as 0.1 nM in the complementary DNA strand to be detected. A similar concept was developed on ZnO coated with a PDA layer to detect and quantify the DNA of the Human Immonodeficiency virus with a detection limit as low as 3.5 pM (Fan et al., 2016). In a similar manner, dual sensing of two analytes can be performed at a single wavelength illumination (Xu et al., 2018). The prostate specific antigen (PSA) can also be quantified from 0.1 pg.mL^−1^ to 20 ng.mL^−1^ using a similar PDA nanoparticles fluorescence quenching assay. The detection limit of PSA was as low as 35 fg.mL^−1^ (Liu et al., 2017).

The PDA nanoparticles (3.1 nm in diameter) synthesized from the solution/air interface by microplasma induced electropolymerization can be used as specific sensors for U^6+^ cations with a detection limit of 2.1 mg.mL^−1^ (Wang et al., 2018). As a similar sensing application, the dopamine oligomers obtained by Xiong et al. (2014) interact in a specific manner with the Fe^3+^ cations (compared to Ag^+^, Ca^2+^, Mg^2+^, Zn^2+^, Cu^2+^, Fe^2+^, Hg^2+^, Pb^2+^, Ni^2+^, Cr^3+^, and Y^3+^). The limit of detection for Fe^3+^ was 0.1 μM and the fluorescence quenching of the dopamine oligomers by the Fe^3+^ cations was linear from 0.1 to 100 μM (Xiong et al., 2014).

Formaldehyde in the gas phase interacts strongly with the PDA through hydrogen bonds with imines present in the PDA film as shown by molecular simulations using the Gaussian software. This concept has been applied to the hollow PDA nanotubes deposited on the surface of a quartz crystal to measure formaldehyde concentrations with a detection limit down to 100 ppb and very low interference with other atmospheric gases, such as H_2_S, CO_2_, and benzene (Yan et al., 2016).

### PDA nanoparticles for theranostic applications

The PDA nanoparticles with an average diameter of 25 to 43 nm were synthesized in reverse emulsions, crosslinked with Fe^3+^ cations, and subsequently modified with poly(ethylene glycol) and used as pH-dependent magnetic resonance imaging contrast agents displaying a high photothermal efficiency owing to the ability of PDA to transform photons in phonons (Liu et al., 2015). The same photon to phonon conversion efficiency is used when the PDA nanoparticles are endocytosed in keratynocytes and undergo subsequent aggregation in their cytosol to form microparosols affording UV protection to those keratynocytes (Huang et al., 2017). The PDA nanoparticles embedded in poly(ethylene glycol) hydrogels afford them some light-triggered responsiveness (Han et al., 2016).

The Gd^3+^ doped PDA nanoparticles display relaxivities of 75 mM^−1^.s^−1^ and 10.3 mM^−1^.s^−1^ at 1.4 T and 7 T, respectively, offering an excellent contrast for magnetic resonance imaging (Wang et al., 2017).

The PDA nanoparticles chelated with Mn^2+^ cations (through surface catechol groups of PDA), and subsequently modified with thiolated PEG, simultaneously offer the possibility to enhance the contrast in magnetic resonance imaging and to ablate cancer cells (HeLa cells) through the photothermal effect afforded by PDA (Miao et al., 2015). Note that the pegylation of these nanoparticles was aimed to simultaneously increase their colloidal stability and their circulation time in the blood stream. The grafting of PEG on PDA@Mn^2+^ almost did not change the Mn^2+^ content per nanoparticle, 1.24 × 10^5^ Mn^2+^ cations /particle, as estimated by the inductively coupled plasma atomic emission spectroscopy.

### PDA nanomaterials for drug release applications

Another major application of the PDA nanoparticles concerns stimulated drug release. PDA nanoparticles around 200 nm in diameter were coated with a porous 5-boronobenzene-1,3-dicarboxylic acid–Zn^2+^ coordination polymer. The obtained core shell nanoparticles were subsequently coated with a catechol capped PEG. These hybrid nanoparticles could be loaded with doxorubicin as a model anticancer drug. The presence of PEG and phenylboronic acid moieties allowed them to target sialic acid overexpressed MCF-7 human breast cancer cells. The acidic environment of such cells allowed for a release of the PEG capping agent and an increased release of doxorubicin. This release was further increased upon irradiation with near infrared (NIR) light (808 nm, about 1 W.cm^−2^) producing a photothermal effect on PDA. The combined photothermal effect and the increased release of doxorubicin was evaluated *in vivo* by measuring the weight of tumors after excision at day 16 following the administration of drug loaded composite nanoparticles and several NIR irradiation steps (Liu et al., 2018). Similarly, co-delivery and photothermal effects were validated with PDA nanoparticles (Xing et al., 2017).

PDA nanoparticles modified with PEG-TTP (TTP is triphenyl phosphonium) could also be loaded with doxorubicin to specifically target the mitochondria in the cells (Li W.-Q. et al., 2017) with the possibility to overcome long-term drug resistance.

Metal organic framework-based particles are ideal porous nanomaterials to load different drugs but their *in vivo* degradation can induce cell apoptosis, organ abnormalities, and even the death of the animals used. The encapsulation of doxorubicin loaded zeolithic imidazole frameworks with a thin PDA layer considerably decreased the decomposition of the metal organic framework without impeding the drug release (Wu et al., 2018).

Composite hollow CaCO_3_-PDA nanoparticles were synthesized by oxidizing a Ca^2+^ salt-dopamine mixture with O_2_ and bubbling NH_4_HCO_3_ in the mixture. These nanoparticles were subsequently modified with PEG and loaded with a photosensitizer, chlorin e6. The fluorescence of this dye was quenched in the presence of PDA that reduced skin photosensitivity but in the acidic environment of cancerous cells, CaCO_3_ was dissolved and the photodynamic effect of the photosensitizer was recovered at the required location (Dong et al., 2018).

The PDA mesoporous nanospheres also offer excellent opportunities for drug release applications as well as for removing dyes such as rhodamine B (Chen et al., 2016).

The PDA nanoparticles display excellent anti-inflammatory properties owing to their eumelanin-like nature affording them the ability to scavenge the reactive oxygen species produced during inflammation. This property was demonstrated in murine models of acute peritonitis and acute lung injury after a single dose of PDA nanoparticles injection (Zhao et al., 2018).

### PDA nanomaterials for tissue engineering

β-Tricalcium phosphate porous scaffolds display excellent affinity for PDA nanoparticles which in turn immobilize various signal biomolecules to promote cellular activity and tissue regeneration (Wang et al., 2016). As perspectives, one could envision the incorporation of the PDA nanoparticles in 3D printed scaffolds where the presence of particles with anti-inflammatory properties could greatly help to guide the immune response of the scaffolds. The imaging contrast afforded by PDA in MRI (Miao et al., 2015) could also help to follow the time evolution of the scaffolds after implantation in a living organism.

## Conclusions and perspectives

PDA can not only find applications as a versatile coating to cover almost all known materials with a conformal layer of a eumelanin-like compound but also as a stable suspension of nanoparticles. These nanoparticles can be produced in the absence of a template, by oxidizing catecholamines in water or in water/ethanol mixtures, and also in the presence of templating molecules. These templating molecules can be surfactants having a considerably long aliphatic chain, as well as polymers, polyelectrolytes, and proteins. The reduction of the hydrodynamic diameter of the PDA nanoparticles is dependent on the concentration of the templating agent. It is of interest to note that the reduction in size of the PDA nanoparticles is accompanied by a progressive decrease, up to total suppression, of the thickness of the PDA coating on the surface of the reaction beaker. This final result suggests a progressive decrease in the amount of free catecholamine able to react with/on surfaces, and correlatively the existence of strong interactions between the catecholamine and the templating molecules. The nature and the strength of the interactions between PDA and templating molecules remain to be investigated in the future to be able to define possible new templating agents for the production of stable inks based on PDA nanoparticles. The structure of the PDA nanoparticles produced in the presence of proteins also remains to be investigated. These particles are biocompatible up to high concentrations and could be either the scrambled egg type with a uniform distribution of the protein in the particle's volume or the core-shell type. Current research is underway in the INSERM 1121 team to elucidate these questions.

However, the PDA based nanoparticles have already found a role in a large set of applications, particularly in the field of biomaterials where they can be used as a bioink and where the ability of PDA to convert light to heat may be fully exploited for cancer treatment by hyperthermy. Many applications in the field of sensing are also emerging owing to the fluorescence quenching ability of PDA. Finally, the PDA loaded scaffolds appear as a material of choice for new applications in the field of tissue engineering.

## Author contributions

The author confirms being the sole contributor of this work and approved it for publication.

### Conflict of interest statement

The author declares that the research was conducted in the absence of any commercial or financial relationships that could be construed as a potential conflict of interest.
